# The association between influenza infection and acute myocardial infarction: A comprehensive systematic review and meta-analysis

**DOI:** 10.1016/j.virusres.2025.199594

**Published:** 2025-06-09

**Authors:** Xia Zhou, Li Feng

**Affiliations:** a Department of Cardiovascular Medicine, People's Hospital of Chongqing Liangjiang New Area (Chongqing Medical University Affiliated Liangjiang Hospital), Chongqing, 401121, China; b Department of Geriatrics, Danzishi Community Health Service Center, Nan 'an District, Chongqing, 400030, China

**Keywords:** Influenza, Acute myocardial infarction, Risk, Outcome, Meta-analysis

## Abstract

Influenza infection could be associated with several systemic complications, including acute myocardial infarction (AMI); however, evidence on this association remains inconsistent. This systematic review and meta-analysis examined the influenza-AMI link, temporal AMI risk post-infection, and in-hospital outcomes and mortality in influenza-infected AMI patients. We conducted a systematic search of PubMed, EMBASE, Cochrane Library, Scopus, and Web of Science. Observational studies and self-controlled case series (SCCS) designs, were included. Data were extracted and analyzed using random-effects models to calculate pooled odds ratios (ORs), incidence rate ratios (IRRs), and 95 % confidence intervals (CIs). Subgroup analyses were performed based on exposure definitions (laboratory-confirmed influenza vs. influenza-like illness [ILI]), study design, and temporal patterns of AMI risk. In-hospital outcomes, including mortality, complications, length of stay, and costs, were also evaluated. The meta-analysis included 17 studies. A significant association was found, with a pooled adjusted OR of 2.70 (95 % CI: 1.28–5.72). ILI showed a stronger association with AMI (aOR: 2.04; 95 % CI: 1.33–3.14) compared to laboratory-confirmed influenza. Temporal analyses from SCCS studies revealed a markedly increased risk of AMI within the first week post-infection, peaking in days 1–3 (IRR: 6.83; 95 % CI: 4.66–10.01) and gradually declining thereafter. Influenza-infected AMI patients had significantly worse in-hospital outcomes, including higher mortality (OR: 1.60; 95 % CI: 1.55–1.66), and multiorgan failure (OR: 2.90; 95 % CI: 2.79–3.01). Additionally, these patients experienced longer median hospital stays (8.8 days vs. 5.5 days) and higher hospitalization costs ($20,678 vs. $18,269) compared to non-influenza AMI patients. This study confirms a strong link between influenza and AMI, especially early post-infection. Influenza-infected AMI patients experience worse outcomes, longer hospital stays, and higher costs. These findings highlight the importance of influenza prevention strategies, including vaccination, particularly in high-risk groups, to reduce AMI risk and its cardiovascular burden.

## Highlights

• We did a meta-analysis on and acute myocardial infarction (AMI).
• Influenza infection was significantly associated with increased risk of AMI.
• A markedly increased risk of AMI was observed within the first week post-infection.
• Influenza-infected AMI patients had significantly worse in-hospital outcomes.
• Influenza-infected AMI patients experienced longer hospital stays and higher costs.

## 1 Introduction

Influenza is an important global health threat, causing 3 to 5 million severe illnesses and 290,000 to 650,000 respiratory deaths every year, resulting in significant economic and healthcare challenges (Hansen et al., 2022; Lafond et al., 2021; WHO, 2019). Besides its known respiratory complications, influenza has systemic effects that may trigger cardiovascular events, including acute myocardial infarction (AMI), a leading cause of death and disability worldwide (Mohammad et al., 2020; Sellers et al., 2017). AMI, characterized by the death of heart muscle tissue due to blood flow obstruction, is usually caused by the rupture of atherosclerotic plaques and following clot formation (Anderson and Morrow, 2017; Thygesen et al., 2018). A recent meta-analysis estimates that the cumulative incidence of AMI associated with laboratory-confirmed influenza infection is 2.19 % (95 % CI: 1.03 %–3.72 %), while the in-hospital mortality rate from influenza-related cardiovascular events is approximately 1.38 % (95 % CI: 0.00 %–4.80 %) (Ouranos et al., 2024). Moreover, some evidence suggests that influenza vaccination may have a protective effect against cardiovascular events, potentially reducing the risk of AMI in vaccinated individuals (Behrouzi et al., 2022; Warren-Gash et al., 2009). Several mechanisms have been linked to influenza-related AMI, including systemic inflammation, endothelial dysfunction, and prothrombotic states, which can result in plaque instability and increased risk of coronary thrombosis (Gopal et al., 2020; Muscente and De Caterina, 2020; Skaarup et al., 2023). These findings indicate the potential importance of understanding the relationship between influenza and AMI to improve prevention strategies.

There is some evidence of a significant association between influenza infection and AMI (Aleem et al., 2023; García-Lledó et al., 2021), but this evidence is inconsistent. Case-control studies have reported mixed results due to selection and recall biases. For example, MacIntyre et al. found that influenza infection did not significantly predict AMI (Macintyre et al., 2013). At the same time, a study in Bangladesh reported no significant relationship between recent respiratory illness and AMI severity (Aleem et al., 2024). Other studies, including Ponka et al. and Warren et al., found no significant association between influenza infection or respiratory illness and AMI (Pönkä et al., 1981; Warren-Gash et al., 2009). Different study designs, definitions of exposure, and population characteristics may contribute to these differences, which suggests that more comprehensive and focused research is needed.

Previous meta-analyses have contributed valuable insights into this topic, yet certain methodological aspects warrant further exploration. For example, Barnes et al. (2015) and Kwok et al. (2015) primarily included observational and case-control studies, without incorporating self-controlled case series (SCCS), a design particularly useful for assessing transient exposures like influenza (Hallas and Pottegård, 2014). Similarly, the meta-analysis by Caldeira et al. (2019) included only three SCCS studies, which may have limited the ability to draw conclusions regarding time-sensitive risks. These considerations highlight the value of an updated analysis that incorporates a broader range of study designs and exposure definitions to strengthen the evidence base.

Based on above-mentioned gaps, the present study aims to conduct an updated systematic review and meta-analysis to evaluate the association between influenza infection and AMI. By incorporating newly available data, and comparing findings across different study designs (SCCS vs. case-control), and examining variations in exposure definitions, this analysis seeks to provide a more comprehensive understanding of this relationship. Additionally, the study evaluates patient outcomes and mortality associated with influenza-related AMI to further inform clinical and public health perspectives.

## 2 Materials and methods

### 2.1 Study design

This systematic review and meta-analysis was conducted by the PRISMA (Preferred Reporting Items for Systematic Reviews and Meta-Analyses) and Cochrane Handbook of Systematic Reviews guidelines (Moher et al., 2009). The primary aim was to evaluate the association between influenza infection and AMI. Observational studies employing various designs, including prospective, case-control and SCCS, were included to compare the risk of AMI following laboratory-confirmed influenza and ILI. This study applies the PECO framework for inclusion criteria, where the population consists of individuals diagnosed with AMI, with or without prior influenza infection. The exposure of interest is laboratory-confirmed influenza or clinically diagnosed ILI. The comparator groups include pre-infection control periods in self-controlled studies and matched non-infected individuals in case-control studies. The primary outcome is the risk of AMI following influenza infection, assessed through effect estimates such as odds ratios, relative risks, or hazard ratios. Secondary outcomes include in-hospital complications such as mortality, cardiogenic shock, respiratory failure, acute kidney injury, and multiorgan failure.

### 2.2 Search strategy

Comprehensive searches were performed in PubMed, EMBASE, Cochrane Library, Scopus, and the Web of Science. The search strategy combined terms such as "influenza infection", "influenza", "flu", "viral infection", "respiratory tract infection", "influenza-like illness", "acute myocardial infarction", "myocardial infarction", "cardiovascular events", and "cardiovascular outcome" using Boolean operators (AND, OR, NOT) to ensure inclusivity. Studies published from inception to 20 February 2025 involving human participants were included. We set no restrictions on geographical area, study design, or language. Studies in languages other than English were translated using Google Translate Tool. Additional searches of gray literature were conducted with Google Scholar (first 30 pages) to minimize publication bias (Chandler et al., 2019). Studies reporting both primary and secondary cardiovascular outcomes following influenza infection were included in the search. The search strategy and MeSH terms/keywords used are presented in Table S1. Investigators further examined reference lists of eligible studies and related reviews to enhance a more comprehensive search.

### 2.3 Selection criteria

We included observational studies evaluating the association between influenza infection (laboratory-confirmed or ILI) and AMI, employing SCCS or case-control designs. Studies must provide sufficient data for effect size estimation, such as odds ratios (OR), relative risks (RR), or hazard ratios (HR) with 95 % confidence intervals (CIs). Additionally, studies reporting secondary outcomes such as mortality, cardiogenic shock, respiratory failure, acute kidney injury, and multiorgan failure were included. Excluded were (1) animal or in vitro studies; (2) studies lacking clear definitions of influenza infection or AMI; (3) non-original articles, including editorials or commentaries; (4) studies on other respiratory infections with lack of data on influenza infection.

### 2.4 Data extraction

Two independent reviewers conducted data extraction using a standardized data collection form, ensuring accuracy and minimizing bias. Discrepancies were resolved through discussion. Extracted data included study characteristics such as study title, authors, year of publication, study period, and country. Information on the study design was collected to classify methodological approaches. Population characteristics, including the mean age of cases and controls, gender ratio, and the income level of the studied countries, were recorded to assess demographic variability. Exposure data were categorized into laboratory-confirmed influenza, determined by specific diagnostic methods, and ILI, based on clinical diagnosis without laboratory confirmation. Data related to the number of cases, controls, and outcome events were extracted to quantify sample sizes. Effect estimates, including crude and adjusted OR, incidence rate ratios (IRR) for different time periods, and the corresponding confidence intervals (CIs), were recorded where available. Adjustments for potential confounders were noted, along with the specific covariates used in each study’s multivariable models. The risk of bias was assessed for each study based on standardized criteria. For self-controlled case-series studies, additional data on the number of episodes and IRR at different time intervals post-influenza infection were collected, specifically examining days 1–3, days 4–7, days 8–14, days 15–28, and days 29–91 to identify temporal variations in AMI risk. We extracted adjusted OR (aOR) where available. Data on in-hospital outcomes, including mortality, cardiogenic shock, acute respiratory failure, acute kidney injury, multiorgan failure, length of hospital stay, and hospitalization costs, were extracted where reported. All extracted data were systematically organized and cross-verified before inclusion in the final meta-analysis.

### 2.5 Risk of bias assessment

Two reviewers independently evaluated the risk of bias in each study. The Newcastle-Ottawa Scale (NOS) was used for case-control studies, assessing selection, comparability, and outcome domains. For SCCS studies, the ROBINS-I (Risk of Bias in Non-Randomized Studies of Interventions) tool was applied, focusing on confounding, participant selection, and outcome measurement (Sterne et al., 2016). Disagreements between reviewers were resolved through discussion to ensure consistency and objectivity in bias assessment.

### 2.6 Statistical analysis

Statistical analyses were performed using R (version 4.1.0) and Stata (version 17) software. Pooled ORs, and IRRs, with 95 % CIs were calculated using a random-effects model to account for between-study heterogeneity (DerSimonian and Laird, 1986). Statistical heterogeneity was assessed using the *I*² statistic and Cochran’s Q test, with thresholds of 25 %, 50 %, and 75 % interpreted as low, moderate, and high heterogeneity, respectively (Higgins and Thompson, 2002). Subgroup analyses were conducted based on exposure definitions (laboratory-confirmed influenza vs. ILI), study implementation period, income levels of countries, study design and risk of bias. Additionally, subgroup analyses were performed according to post-influenza time intervals, including days 1–3, days 4–7, days 8–14, days 15–28, and days 1–28, to evaluate temporal patterns of AMI risk following infection. Cumulative meta-analyses were applied to determine the reliability of the estimated ORs. Secondary analyses were performed to assess in-hospital outcomes, including mortality, cardiogenic shock, respiratory failure, acute kidney injury, and multiorgan failure, to determine the clinical impact of influenza-related AMI. Funnel plots were visually inspected, and Egger’s test was conducted to assess publication bias (Egger et al., 1997). Sensitivity analyses were performed by excluding studies with a high risk of bias to evaluate the robustness of the findings.

## 3 Results

### 3.1 Study selection and characteristics

**Fig. 1:**
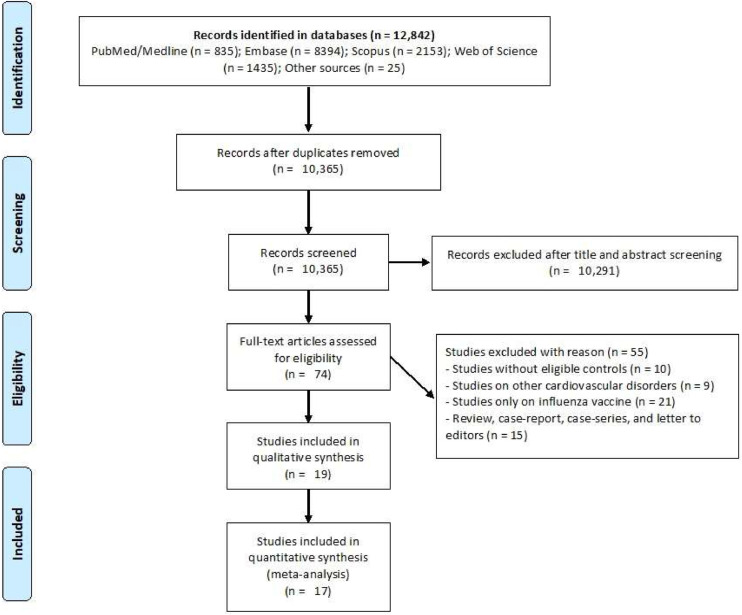
PRISMA flowchart of study selection process.

A total of 12,824 studies were identified through database searches, with 10,365 remaining after duplicate removal. Following title and abstract screening, 10,291 records were excluded, leaving 74 studies for full-text evaluation. Of these, 19 studies (comprising 19 datasets) met the inclusion criteria (Aleem et al., 2023; Cardoso et al., 2020; Cheng et al., 2022; de Boer et al., 2024; Guan et al., 2012; Korves et al., 2024; Kwong et al., 2018; Macintyre et al., 2013; Mattila, 1989; Mohammad et al., 2020; Mohseni et al., 2024; Pönkä et al., 1981; Tripathi et al., 2020; Vejpongsa et al., 2019; Warren-Gash et al., 2018, 2013, 2012; Yang and Xu, 2023; Young-Xu et al., 2020). The meta-analysis included 17 studies (Fig. 1) (Aleem et al., 2023; Cardoso et al., 2020; Cheng et al., 2022; de Boer et al., 2024; Guan et al., 2012; Korves et al., 2024; Kwong et al., 2018; Macintyre et al., 2013; Mattila, 1989; Mohseni et al., 2024; Pönkä et al., 1981; Tripathi et al., 2020; Vejpongsa et al., 2019; Warren-Gash et al., 2018, 2013, 2012; Yang and Xu, 2023), as well as two studies considered as part of the systematic review (Mohammad et al., 2020; Young-Xu et al., 2020). Nine observational studies provided 12 datasets that examined the association between influenza infection and AMI (Aleem et al., 2023; Cheng et al., 2022; Guan et al., 2012; Macintyre et al., 2013; Mattila, 1989; Mohseni et al., 2024; Pönkä et al., 1981; Warren-Gash et al., 2013; Yang and Xu, 2023). There were seven SCCS studies that evaluated IRRs (de Boer et al., 2024; Korves et al., 2024; Kwong et al., 2018; Mohammad et al., 2020; Warren-Gash et al., 2018, 2012; Young-Xu et al., 2020), five of which contributed to the meta-analysis (de Boer et al., 2024; Korves et al., 2024; Kwong et al., 2018; Warren-Gash et al., 2018, 2012) and two of which contributed only to the systematic review (Mohammad et al., 2020; Young-Xu et al., 2020). Three studies examined in-hospital outcomes among AMI patients with influenza (Cardoso et al., 2020; Tripathi et al., 2020; Vejpongsa et al., 2019). These studies were conducted across different geographical and economic conditions, providing a variety of epidemiological perspectives. In the following tables, detailed study characteristics are provided.

The risk of bias was assessed using the Newcastle-Ottawa Scale for case-control studies and the ROBINS-I tool for self-controlled designs. Although two studies were classified as having a high bias risk, the majority showed a moderate (*n* = 3) to low risk (*n* = 12).

### 3.2 Association between influenza infection and acute myocardial infarction

The association between influenza infection and AMI was examined in nine observational studies with 12 datasets (Aleem et al., 2023; Cheng et al., 2022; Guan et al., 2012; Macintyre et al., 2013; Mattila, 1989; Mohseni et al., 2024; Pönkä et al., 1981; Warren-Gash et al., 2013; Yang and Xu, 2023). Of these, six datasets relied on laboratory-confirmed influenza (Aleem et al., 2023; Guan et al., 2012; Macintyre et al., 2013; Pönkä et al., 1981; Warren-Gash et al., 2013), while six datasets used an ILI definition (Cheng et al., 2022; Mattila, 1989; Mohseni et al., 2024; Pönkä et al., 1981; Warren-Gash et al., 2013; Yang and Xu, 2023). In all studies, participants were older than 40 years. There were five prospective hospital-based investigations (Guan et al., 2012; Macintyre et al., 2013; Mattila, 1989; Pönkä et al., 1981; Warren-Gash et al., 2013), and four retrospective case-control studies (Aleem et al., 2023; Cheng et al., 2022; Mohseni et al., 2024; Yang and Xu, 2023). Different studies used different laboratory methods. Quantitative real-time polymerase chain reaction (qRT-PCR) was used in one study (Aleem et al., 2023), IgA antibody testing was performed in another (Warren-Gash et al., 2013), and serological tests were used in the remaining studies, including one that assessed both influenza A and B (Guan et al., 2012). ILI diagnoses were determined by the International Classification of Disease (ICD) codes. Detailed characteristics of these observational studies are presented in Table S2.

**Fig. 2:**
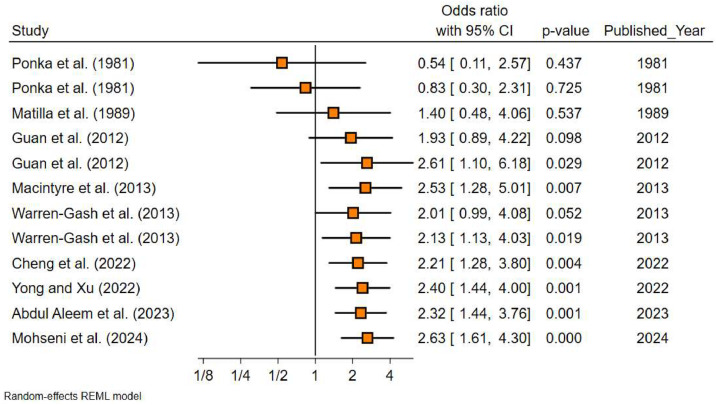
Cumulative meta-analysis of the association between influenza infection and AMI. The ORs with 95 % CIs are displayed for each study, arranged chronologically by publication year.

The pooled analysis revealed a statistically significant association between influenza infection and AMI. The overall crude OR was 2.63 (95 % CI: 1.61–4.30; *I*^2^ = 78.7), while the adjusted OR was 2.70 (95 % CI: 1.28–5.72; *I*^2^ = 79.1) (Table 1). In the subgroup analysis, studies focusing on laboratory-confirmed influenza did not show statistical significance, with an adjusted OR of 2.92 (95 % CI: 0.87–9.82). In contrast, ILI demonstrated a significant association, with an adjusted OR of 2.04 (95 % CI: 1.33–3.14). Regarding the study period, studies conducted between 2011 and 2024 showed a significant association (aOR: 2.01; 95 % CI: 1.31–3.09), whereas studies conducted before 2010 did not. Notably, studies from middle- and lower-middle-income countries reported a significantly higher risk estimate (aOR: 5.92; 95 % CI: 2.17–16.18), while studies from high-income countries did not find a significant association (aOR: 1.39; 95 % CI: 0.89–2.15). Considering the risk of bias, studies with a moderate risk of bias demonstrated a statistically significant association (aOR: 7.23; 95 % CI: 2.23–23.51), whereas studies with a low risk of bias did not (aOR: 1.45; 95 % CI: 0.96–2.17) (Table 1). The cumulative meta-analysis reveals a progressive increase in the OR linking influenza infection to AMI over time. Early studies showed inconsistent associations, with wide CIs and non-significant association. However, more recent studies (2012–2024) indicate a stronger and more statistically significant association (Fig. 2). There was no publication bias in included studies (β= −1.82, *p* = 0.161) (Fig. S1).

**Table 1:** Pooled results for analysis of studies evaluating Influenza infection and risk of AMI.

Variables	Crude OR	Adjusted OR
**Overall**	2.63 (1.61–4.30)	2.70 (1.28–5.72)
**Influenza definition**		
Laboratory-confirmed influenza	1.90 (0.86–4.20)	2.92 (0.87–9.82)
Influenza-Like symptoms	3.71 (2.12–6.47)	2.04 (1.33–3.14)
**Study year period**		
<2000	1.40 (0.48–4.06)	–
2000–2010	2.55 (1.15–5.69)	3.03 (0.90–10.16)
2011–2024	3.88 (1.89–7.96)	2.01 (1.31–3.09)
**Income of countries**		
High	1.66 (1.01–2.76)	1.39 (0.89–2.15)
Middle of lower-middle	5.04 (3.01–8.45)	5.92 (2.17–16.18)
**Study design**		
Prospective hospital-based study	2.13 (1.13–4.03)	3.03 (0.90–10.16)
Retrospective case-control	3.88 (1.89–7.96)	2.01 (1.31–3.09)
**Risk of bias**		
High	1.40 (0.48–4.06)	–
Moderate	5.88 (3.72–9.29)	7.23 (2.23–23.51)
Low	1.72 (0.99–2.98)	1.45 (0.96–2.17)

### 3.3 AMI risk after influenza infection: time patterns

We included five SCCS studies in our meta-analysis (de Boer et al., 2024; Korves et al., 2024; Kwong et al., 2018; Warren-Gash et al., 2018, 2012), four of which evaluated laboratory-confirmed influenza and one that assessed influenza-like illness. The characteristics of these studies are summarized in Table S3. Time-stratified analyses revealed a strong temporal association between laboratory-confirmed influenza and AMI risk (Table 2). The cumulative IRR of AMI within days 1–7 post-infection was 6.77 (95 % CI: 5.84–7.86), with the highest IRR observed within the first 1–3 days (IRR: 6.83; 95 % CI: 4.66–10.01), followed by days 4–7 (IRR: 5.24; 95 % CI: 3.55–7.75). The risk gradually declined over time, with IRRs of 1.89 (95 % CI: 1.43–2.51) for days 8–14 and 1.68 (95 % CI: 1.32–2.15) for days 15–28. The cumulative IRR over the first 28 days post-infection was 3.52 (95 % CI: 3.10–3.40) (Table 2). Heterogeneity for this meta-analysis was zero.

**Table 2:** Pooled results for analysis of incidence ratios for acute myocardial infarction after laboratory-confirmed influenza infection.

Time period (days)	Number of datasets	IRR (95 % CI)
1–7	3	6.77 (5.84–7.86)
1–3	4	6.83 (4.66–10.01)
4–7	4	5.24 (3.55–7.75)
8–14	5	1.89 (1.43–2.51)
15–28	5	1.68 (1.32–2.15)
1–28	2	3.52 (3.10–3.40)

Two SCCS studies were excluded from the meta-analysis due to methodological differences. Mohammad et al. (2020) examined the weekly incidence of MI relative to influenza case counts, stratified into tertiles of 0–16, 17–164, and more than 164 cases per week. Weeks with 0–16 reported cases showed no significant association with AMI incidence after adjusting for weather parameters (adjusted IRR: 1.03; 95 % CI: 1.00–1.06; *P* = 0.09). However, higher influenza case counts were significantly associated with increased AMI incidence. Specifically, 17–164 cases per week yielded an adjusted IRR of 1.05 (95 % CI: 1.02–1.08; *P* = 0.003), and more than 164 cases per week yielded an adjusted IRR of 1.06 (95 % CI: 1.02–1.09; *P* = 0.002). Young-Xu et al. (2020) analyzed AMI risk within one year before and after a positive influenza test, identifying 391 hospitalizations for AMI. Of these, 31 occurred during the risk interval (31.1 admissions per week) and 360 during the control interval (3.5 admissions per week), yielding an incidence ratio (IR) of 8.89 (95 % CI: 6.16–12.84). Stratified analyses revealed significantly elevated AMI risk among patients with high white blood cell counts (IR: 12.43; 95 % CI: 6.99–22.10) and high platelet counts (IR: 15.89; 95 % CI: 3.59–70.41).

### 3.4 In-hospital outcomes in AMI patients with concomitant influenza

All three studies on in-hospital outcomes were conducted in the United States (Table S4). Patients hospitalized with AMI and co-existing influenza experienced significantly worse clinical outcomes (Table 3). The odds of in-hospital mortality were 1.60 times higher (95 % CI: 1.55–1.66) among influenza-infected AMI patients compared to those without influenza. Additionally, influenza-infected AMI patients had increased odds of acute respiratory failure (OR: 3.90, 95 % CI: 3.48–4.37), acute kidney injury (OR: 2.30, 95 % CI: 2.04–2.59), and multiorgan failure (OR: 2.90, 95 % CI: 2.79–3.01). The association with cardiogenic shock did not reach statistical significance (OR: 1.45, 95 % CI: 0.94–2.25). Moreover, influenza-infected AMI patients had a longer median hospital stay (8.8 ± 10.1 vs. 5.5 ± 7.14 days) and higher hospitalization costs (20,678±3781$ vs. 18,269.5 ± 2918.9$) compared to those without influenza.

**Table 3:** Pooled results for analysis of In-hospital outcomes for acute myocardial infarction patients with concomitant influenza infection as compared to those without influenza.

Outcome	Patients with influenza	Patients with outcome	People without influenza	Patients with outcome	OR (95 % CI)
In-hospital mortality	25,721	3237	8561,781	684,906	1.60 (1.55–1.66)
Cardiogenic shock	25,721	2838	8561,781	562,896	1.45 (0.94–2.25)
Acute respiratory failure (ARF)	25,721	13,381	8561,781	1811,932	3.90 (3.48–4.37)
ARF requiring mechanical ventilation	15,836	4876	6698,166	690,875	2.78 (1.66–4.66)
Acute kidney injury (AKI)	25,721	10,043	8561,781	1798,919	2.30 (2.04–2.59)
AKI requiring dialysis	22,715	974	6136,426	79,002	3.24 (2.63–4.01)
Multiorgan failure	12,830	4251	4272,811	624,203	2.90 (2.79–3.01)
Length of stay, median (days)	22,715	8.8 ± 10.1	8561,781	5.5 ± 7.14	1.83 (1.045 - 3.20)
Hospital costs, median ($)	22,715	20,678±3781	6698,166	18,269.5 ± 2918.9	2408.5∗∗ (1472.2- 3344.8)

∗∗ mean difference.

## 4 Discussion

Our systematic review and meta-analysis of observational studies demonstrated a statistically significant association between influenza infection and AMI. Notably, when stratified by exposure definition, studies using an ILI criterion showed a stronger and statistically significant association, whereas those relying on laboratory-confirmed influenza did not reach statistical significance. Additionally, subgroup analyses revealed that studies conducted in middle- and lower-middle-income countries reported higher risk estimates compared to those from high-income regions. Furthermore, time‐stratified analyses of SCCS studies reveal that the risk of AMI peaks within 1–3 days post-infection—with an IRR of 6.83—and gradually declines over time intervals, indicating a critical period of infection-induced inflammation and prothrombosis. In addition to the primary association between influenza and AMI, our analysis of in-hospital outcomes highlights the substantial clinical impact of influenza among patients presenting with AMI. To our knowledge, this is the first meta-analysis that assesses these in-hospital outcomes, demonstrating that patients with concomitant influenza infection experienced significantly worse outcomes compared to non-infected individuals. Specifically, the odds of in-hospital mortality were 1.60 times higher, and these patients had increased odds of complications such as acute respiratory failure, acute kidney injury, and multiorgan failure. Moreover, influenza-infected AMI patients exhibited a longer median hospital stay (8.8 days versus 5.5 days) and incurred higher hospitalization costs ($20,678 versus $18,269). These findings suggest that influenza not only triggers AMI but also exacerbates the severity of the acute event and the associated healthcare burden.

Previous meta-analyses of observational studies support our finding of a statistically significant association between influenza infection and AMI. In a meta-analysis of case-control studies, Barnes et al. found that recent respiratory infections, ILI, or respiratory tract infections were significantly more likely to occur in AMI cases (Barnes et al., 2015). AMI was significantly associated with studies of ILI (OR 2.29, 95 % CI 1.11 to 4.73), whereas laboratory-diagnosed influenza studies were not significant (OR 2.44, 95 % CI 0.83 to 7.20) (Barnes et al., 2015). Similarly, Kwok et al. in another meta-analysis found a pooled OR for risk of MI with influenza-like symptoms of 2.17 (95 % CI 1.68–2.80), but the risk was less evident with serologically defined influenza (Kwok et al., 2015).

The stronger association between ILI and AMI compared to laboratory-confirmed influenza may be attributed to several factors. As a first point, ILI definitions, which rely on clinical symptoms such as fever, cough, and sore throat, cover a wide range of respiratory infections, including parainfluenza, respiratory syncytial virus (RSV), and coronavirus, which can cause similar symptoms, which can trigger inflammatory responses (Huo et al., 2012; Vassalle, 2023; Warren-Gash et al., 2009). This non-specificity introduces the possibility of misclassification bias and will inflate the observed AMI association by including infections other than influenza. But this broader clinical categorization may also reflect the real-world burden of acute respiratory illness on cardiovascular health. In contrast, laboratory confirmed studies will underestimate the association because they will include asymptomatic or mildly symptomatic cases (Kwok et al., 2015) with lower inflammatory burden or because of diagnostic limitations related to timing and test sensitivity. Supporting this, recent studies help clarify why ILI may show a stronger signal. Mohseni et al. (2024) observed a significantly higher risk of AMI in patients with ILI, likely because this clinical definition aligns closely with peak symptom presentation and inflammatory activity. In contrast, Young-Xu et al. (2020) found that among laboratory-confirmed influenza cases, the elevated AMI risk was most pronounced in individuals with high inflammatory markers—again pointing to the role of inflammation rather than viral presence alone. These findings suggest that diagnostic scope, symptom timing, and host inflammatory response all play a part in shaping the observed association with AMI. Additionally, technical limitations such as the narrow detection window of RT-PCR (typically within 2–3 days of symptom onset) and the retrospective nature of serological testing, which may reflect past rather than acute infections, further reduce the sensitivity of laboratory-confirmed approaches (Guan et al., 2012; Lau et al., 2010; Muscente and De Caterina, 2020). Additionally, the symptomatic phase captured by ILI is more closely associated with the strong inflammatory response that triggers AMI, characterized by plaque destabilization, platelet activation, and increased metabolic demand. Lastly, studies using laboratory-confirmed criteria may have lower statistical power due to fewer confirmed cases, thereby making it harder to detect a significant association (Barnes et al., 2015; Macintyre et al., 2013). We also recognize that some subgroup estimates, such as those involving lab-confirmed influenza or studies with low risk of bias, were accompanied by wide confidence intervals. These broader intervals likely reflect smaller sample sizes or fewer events in these categories, limiting the precision of effect estimates. This kind of imprecision is not uncommon in meta-analyses with heterogeneous data, and we encourage cautious interpretation of these specific findings in light of their statistical uncertainty (Caldeira et al., 2019). As a result of these factors, ILI definitions identify a wider, clinically relevant spectrum of respiratory infections that better reflect the inflammatory environment that leads to AMI.

The subgroup analysis revealed significantly higher risk estimates for influenza-associated AMI in middle- and lower-middle-income countries (aOR: 5.92) compared to high-income countries (aOR: 1.39). This disparity may reflect several contextual factors. Reduced access to timely and high-quality healthcare can lead to delays in the diagnosis and management of both influenza and AMI, potentially exacerbating the clinical outcomes. Moreover, lower vaccination coverage in these regions increases susceptibility to influenza and its complications. The higher burden of untreated or undiagnosed comorbid conditions, such as diabetes, hypertension, and chronic lung disease, may also elevate baseline cardiovascular risk. Additionally, differences in diagnostic capacity and reporting infrastructure may contribute to overestimation of associations in these settings due to selective case detection or residual confounding. These contextual factors should be considered when interpreting regional variations in effect size.

Caldeira et al. reported similar results to our findings based on SCSS studies. In their findings (Caldeira et al., 2019), the IRR was 5.79 in the first three days following an influenza infection and 4.52 in the period 4–7 days following an influenza infection. However, the risk was not significantly increased in 8–14 and 15–28 days. Ohland et al. identified markedly elevated IRs of myocardial infarction after respiratory infections, further emphasizing the acute nature of the risk (Ohland et al., 2020). Ruane et al. in another study showed a 17-fold increased risk of MI within 7 days of respiratory infection symptoms, where the relative risk gradually decreased but remained elevated one month after respiratory infection (Ruane et al., 2017).The overall findings from SCCS studies provide strong evidence that the period immediately following an influenza infection represents a critical window of higher risk for AMI.

The underlying pathophysiological mechanisms further support our findings. Influenza infection triggers an intense systemic inflammatory response characterized by the release of pro-inflammatory cytokines such as interleukins (IL-1, IL-6, IL-8), tumor necrosis factor-alpha, and interferons, which activate inflammatory cells within atherosclerotic plaques (Bocale et al., 2022; Gopal et al., 2020). This systemic inflammation increases the production of metalloproteinases and reactive oxygen species, destabilizing plaques, and contributing to endothelial dysfunction through the upregulation of adhesion molecules that facilitate leukocyte infiltration (Haidari et al., 2010; Wang et al., 2010; Zeng et al., 2012). Furthermore, there is evidence that influenza viruses can directly infect vascular endothelial cells and atherosclerotic plaques, further triggering local inflammatory responses (Musher et al., 2019). Influenza also promotes thrombus formation at sites of plaque rupture by enhancing platelet activation, coagulation, and reducing fibrinolysis (Levi et al., 2003; Naghavi et al., 2003; Vallance et al., 1997). Finally, hemodynamic changes induced by the infection—including tachycardia, hypoxia, and changes in blood flow—increase myocardial oxygen demand and may lead to ischemia (Bocale et al., 2022; Muscente and De Caterina, 2020; Musher et al., 2019). Other mechanisms, though less common, may also play a role. Direct myocardial injury, such as myocarditis, can occur following influenza infection (Chughtai et al., 2020), and the virus may indirectly impact cardiovascular risk by impairing the anti-inflammatory functions of high-density lipoproteins (HDL), thereby promoting the oxidation of low-density lipoprotein (LDL) and furthering inflammation (Hebsur et al., 2014; Navab et al., 2003). Furthermore, the infection may interact with other pathogens or modulate immune responses to them, which could contribute to the progression of atherosclerosis (Naghavi et al., 2000).

Our study has several notable strengths. We enhanced the reliability and generalizability of our findings by including multiple datasets and subgroup analyses based on exposure type, geographical region, and time interval. In addition, the detailed assessment of in-hospital outcomes provides valuable clinical insights into the impact of concomitant influenza infection in AMI patients. There are, however, some limitations to be considered. Laboratory-confirmed influenza and ILI have different diagnostic criteria, which introduces heterogeneity. Due to the observational nature of the included studies, the analysis is susceptible to residual confounding and selection biases. The pooled results may not be generalizable due to differences in population demographics, healthcare settings, and methodological approaches across studies, and even though gray literature has been included to reduce publication bias, its influence cannot be completely eliminated. Finally, the meta-analysis could not fully adjust for country-level confounders such as vaccination rates, healthcare infrastructure, or environmental exposures due to limited primary data. Future studies should stratify analyses by these factors to clarify their role in the observed disparities.

In conclusion, our meta-analysis provides strong evidence that influenza infection significantly increases the risk of AMI, particularly in the immediate days following infection. The strong association observed with ILI definitions and the strong time pattern evident in SCCS studies highlight the clinical relevance of the inflammatory and prothrombotic responses induced by the infection. Importantly, the adverse in-hospital outcomes observed among AMI patients with concomitant influenza—such as increased mortality, higher complication rates, prolonged hospital stays, and elevated costs—emphasize the critical need for effective influenza prevention and early intervention strategies in populations at elevated cardiovascular risk. In the future, research should focus on understanding the underlying pathophysiological mechanisms and assessing whether targeted interventions can reduce both influenza-associated cardiovascular events' incidence and severity. Our findings also carry important public health suggestions. Expanding influenza vaccination programs—especially among middle-aged adults with cardiovascular risk—could help reduce not just flu-related illness but also secondary events like AMI. A recent analysis by Raj et al. (2019) suggested that such a strategy could prevent over 1400 AMI hospitalizations each year and yield significant cost savings. These results highlight the broader cardiovascular benefits of influenza prevention and support incorporating AMI risk reduction into vaccine policy discussions.

## Abbreviations

The following abbreviations are used in this manuscript:

**Table:** 

AMI	Acute myocardial infarction
SCCS	Self-controlled case series
ORs	Odds ratios
CIs	Confidence intervals
ILI	Influenza-like illness
IRRs	Incidence rate ratios

## Institutional review board statement

Not applicable.

## Informed consent statement

Not applicable.

## Funding

This research received no external funding.

## CRediT authorship contribution statement

**Xia Zhou:** Writing – review & editing, Writing – original draft, Visualization, Validation, Software, Methodology, Investigation, Formal analysis, Data curation, Conceptualization. **Li Feng:** Writing – review & editing, Writing – original draft, Visualization, Validation, Supervision, Software, Resources, Project administration, Methodology, Investigation, Formal analysis, Data curation, Conceptualization.

## Declaration of competing interest

The authors declare that they have no known competing financial interests or personal relationships that could have appeared to influence the work reported in this paper.

## Data Availability

All data is included in the manuscript or supplementary files.

## Appendix Supplementary materials

Image, application 1

## References

[bib0001] Aleem M.A. (2024). Prevalence of influenza and other acute respiratory illnesses in patients with acute myocardial infarction in Bangladesh: a cross-sectional study. Health Sci. Rep..

[bib0002] Aleem M.A. (2023). Association of recent respiratory illness and influenza with acute myocardial infarction among the Bangladeshi population: a case-control study. Epidemiol. Infect..

[bib0003] Anderson J.L., Morrow D.A. (2017). Acute myocardial infarction. New Engl. J. Med..

[bib0004] Barnes M. (2015). Acute myocardial infarction and influenza: a meta-analysis of case-control studies. Heart.

[bib0005] Behrouzi B. (2022). Association of influenza vaccination with cardiovascular risk: a meta-analysis. JAMA Netw. Open.

[bib0006] Bocale R., Necozione S., Desideri G. (2022). The link between influenza and myocardial infarction: vaccination protects. Eur. Heart. J..

[bib0007] Caldeira D. (2019). The association of influenza infection and vaccine with myocardial infarction: systematic review and meta-analysis of self-controlled case series. Expert Rev. Vaccines.

[bib0008] Cardoso R. (2020). In-hospital management and outcomes of patients with acute myocardial infarction and influenza. Am. J. Cardiol..

[bib0009] Chandler J. (2019).

[bib0010] Cheng H.Y. (2022). Early risk of acute myocardial infarction following hospitalization for severe influenza infection in the middle-aged population of Hong Kong. PloS One.

[bib0011] Chughtai A.A. (2020). Association of influenza infection and vaccination with cardiac biomarkers and left ventricular ejection fraction in patients with acute myocardial infarction. Int. J. Cardiol. Heart. Vasc..

[bib0012] de Boer A.R. (2024). Influenza infection and acute myocardial infarction. NEJM Evid..

[bib0013] DerSimonian R., Laird N. (1986). Meta-analysis in clinical trials. Control. Clin. Trials.

[bib0014] Egger M. (1997). Bias in meta-analysis detected by a simple, graphical test. BMJ.

[bib0015] García-Lledó A. (2021). Relationship between influenza, temperature, and type 1 myocardial infarction: an ecological time-series study. J. Am. Heart Assoc..

[bib0016] Gopal R., Marinelli M.A., Alcorn J.F. (2020). Immune mechanisms in cardiovascular diseases associated with viral infection. Front. Immunol..

[bib0017] Guan X. (2012). Association of influenza virus infection and inflammatory cytokines with acute myocardial infarction. Inflamm. Res..

[bib0018] Haidari M. (2010). Influenza virus directly infects, inflames, and resides in the arteries of atherosclerotic and normal mice. Atherosclerosis.

[bib0019] Hallas J., Pottegård A. (2014). Use of self-controlled designs in pharmacoepidemiology. J. Intern. Med..

[bib0020] Hansen C.L. (2022). Mortality associated with influenza and respiratory syncytial virus in the US, 1999-2018. JAMA Netw. Open.

[bib0021] Hebsur S. (2014). Influenza and coronary artery disease: exploring a clinical association with myocardial infarction and analyzing the utility of vaccination in prevention of myocardial infarction. Rev. Cardiovasc. Med..

[bib0022] Higgins J.P., Thompson S.G. (2002). Quantifying heterogeneity in a meta-analysis. Stat. Med..

[bib0023] Huo X. (2012). Surveillance of 16 respiratory viruses in patients with influenza-like illness in Nanjing, China. J. Med. Virol..

[bib0024] Korves C. (2024). Coronary and cerebrovascular events and exacerbation of existing conditions after laboratory-confirmed influenza infection among US veterans: a self-controlled case series study. Influenza Other Respir. Viruses.

[bib0025] Kwok C.S. (2015). Influenza, influenza-like symptoms and their association with cardiovascular risks: a systematic review and meta-analysis of observational studies. Int. J. Clin. Pract..

[bib0026] Kwong J.C. (2018). Acute myocardial infarction after laboratory-confirmed influenza infection. New Engl. J. Med..

[bib0027] Lafond K.E. (2021). Global burden of influenza-associated lower respiratory tract infections and hospitalizations among adults: a systematic review and meta-analysis. PLoS Med..

[bib0028] Lau L.L. (2010). Viral shedding and clinical illness in naturally acquired influenza virus infections. J Infect. Dis..

[bib0029] Levi M., Keller T.T., van Gorp E., ten Cate H. (2003). Infection and inflammation and the coagulation system. Cardiovasc. Res..

[bib0030] Macintyre C.R. (2013). Ischaemic heart disease, influenza and influenza vaccination: a prospective case control study. Heart.

[bib0031] Mattila K.J. (1989). Viral and bacterial infections in patients with acute myocardial infarction. J. Int. Med..

[bib0032] Mohammad M.A. (2020). Association of acute myocardial infarction with influenza: a nationwide observational study. PloS One.

[bib0033] Moher D. (2009). Preferred reporting items for systematic reviews and meta-analyses: the PRISMA statement. PLoS Med..

[bib0034] Mohseni S.M. (2024). Association between Influenza-like illness and acute myocardial infarction patients: a case-control study. Authorea Preprints.

[bib0035] Muscente F., De Caterina R. (2020). Causal relationship between influenza infection and risk of acute myocardial infarction: pathophysiological hypothesis and clinical implications. Eur. Heart J..

[bib0036] Musher D.M., Abers M.S., Corrales-Medina V.F. (2019). Acute infection and myocardial infarction. New Engl. J. Med..

[bib0037] Naghavi M. (2000). Association of influenza vaccination and reduced risk of recurrent myocardial infarction. Circulation.

[bib0038] Naghavi M. (2003). Influenza infection exerts prominent inflammatory and thrombotic effects on the atherosclerotic plaques of apolipoprotein E-deficient mice. Circulation.

[bib0039] Navab M. (2003). Human apolipoprotein AI mimetic peptides for the treatment of atherosclerosis. Curr. Opin. Investig. Drugs.

[bib0040] Ohland J. (2020). Acute myocardial infarctions and stroke triggered by laboratory-confirmed respiratory infections in Denmark, 2010 to 2016. Eurosurveillance.

[bib0041] Ouranos K. (2024). Cumulative incidence and mortality rate of cardiovascular complications due to laboratory-confirmed influenza virus infection: a systematic review and meta-analysis. Rev. Med. Virol..

[bib0042] Pönkä A. (1981). Viral and mycoplasmal antibodies in patients with myocardial infarction. Ann. Clin. Res..

[bib0043] Raj S.M. (2019). The impact of cardiovascular prevention on the cost benefit of influenza vaccination for australian adults aged 50–64 years old. Heart Lung Circ..

[bib0044] Ruane L. (2017). Triggering of acute myocardial infarction by respiratory infection. Int. Med. J..

[bib0045] Sellers S.A., Hagan R.S., Hayden F.G., Fischer W.A. (2017). The hidden burden of influenza: a review of the extra-pulmonary complications of influenza infection. Influenza Other Respir. Viruses.

[bib0046] Skaarup K.G. (2023). Influenza and cardiovascular disease pathophysiology: strings attached. Eur. Heart J..

[bib0047] Sterne J.A. (2016). ROBINS-I: a tool for assessing risk of bias in non-randomised studies of interventions. BMJ.

[bib0048] Thygesen K. (2018). Fourth universal definition of myocardial infarction (2018). J. Am. Coll. Cardiol..

[bib0049] Tripathi B. (2020). Influence of influenza infection on in-hospital acute myocardial infarction outcomes. J. Am. Cardiol..

[bib0050] Vallance P., Collier J., Bhagat K. (1997). Infection, inflammation, and infarction: does acute endothelial dysfunction provide a link?. Lancet.

[bib0051] Vassalle C. (2023). Viral infections in cardiometabolic risk and disease between old acquaintances and new enemies. Explor. Cardiol..

[bib0052] Vejpongsa P. (2019). Outcomes of acute myocardial infarction in patients with influenza and other viral respiratory infections. Am. J. Med..

[bib0053] Wang S. (2010). Influenza virus-cytokine-protease cycle in the pathogenesis of vascular hyperpermeability in severe influenza. J. Infect. Dis..

[bib0054] Warren-Gash C. (2018). Laboratory-confirmed respiratory infections as triggers for acute myocardial infarction and stroke: a self-controlled case series analysis of national linked datasets from Scotland. Eur. Respir. J..

[bib0055] Warren-Gash C. (2013). Influenza-like illness in acute myocardial infarction patients during the winter wave of the influenza A H1N1 pandemic in London: a case-control study. BMJ Open.

[bib0056] Warren-Gash C. (2012). Influenza infection and risk of acute myocardial infarction in England and Wales: a CALIBER self-controlled case series study. J. Infect. Dis..

[bib0057] Warren-Gash C., Smeeth L., Hayward A.C. (2009). Influenza as a trigger for acute myocardial infarction or death from cardiovascular disease: a systematic review. Lancet Infect. Dis..

[bib0058] World Health Organization (WHO), 2019. Global Influenza Strategy 2019-2030.

[bib0059] Yang X., Xu X. (2023). Evaluation of effect of influenza-like virus in adults: a case control study on adults with myocardial infarction problems. Pak. J. Zool..

[bib0060] Young-Xu Y. (2020). Laboratory-confirmed influenza infection and acute myocardial infarction among United States senior Veterans. PloS One.

[bib0061] Zeng H. (2012). Human pulmonary microvascular endothelial cells support productive replication of highly pathogenic avian influenza viruses: possible involvement in the pathogenesis of human H5N1 virus infection. J. Virol..

