# Supplementary material for: The association between influenza infection and acute myocardial infarction: A comprehensive systematic review and meta-analysis
Source: Virus Res. 2025 Jun 9;358:199594. doi: 10.1016/j.virusres.2025.199594 (PMC12221650; doi:10.1016/j.virusres.2025.199594)
Supplement: Supplementary file 1 [file mmc1.docx]

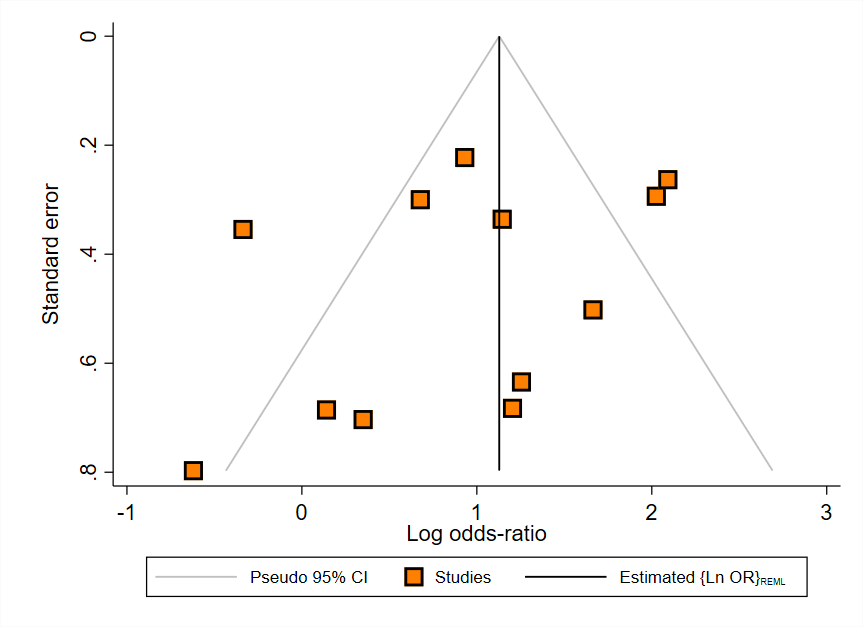


**Figure S1.** Funnel plot assessing publication bias in the meta-analysis of the association between influenza infection and acute myocardial infarction (AMI). The distribution of studies around the pooled effect estimate is examined for asymmetry, which may indicate potential publication bias.

**Supplementary Table 1.** Search strategy in databases

| Database | Limits | Descriptors | Number of studies reached |
| --- | --- | --- | --- |
| PubMed | - | ("influenza infection"[All Fields] OR "influenza"[All Fields] OR "flu"[All Fields] OR "viral infection"[All Fields] OR "respiratory tract infection"[All Fields] OR "influenza-like illness"[All Fields]) AND ("acute myocardial infarction"[All Fields] OR "myocardial infarction"[All Fields] OR "cardiovascular events"[All Fields] OR "cardiovascular outcome"[All Fields]) | 835 |
| Web of Science | Article | (TOPIC: ("influenza infection" OR "influenza" OR "flu" OR "viral infection" OR "respiratory tract infection" OR "influenza-like illness") AND ("acute myocardial infarction" OR ("myocardial infarction" OR "cardiovascular events" OR "cardiovascular outcome")) | 1,435 |
| Scopus | Article | TITLE-ABS-KEY ((“influenza infection" OR "influenza" OR "flu" OR "viral infection" OR "respiratory tract infection" OR "influenza-like illness”) AND ("acute myocardial infarction" OR "myocardial infarction" OR "cardiovascular events" OR "cardiovascular outcome")) | 2153 |
| Embase, | - | ('influenza infection'/exp OR 'influenza infection' OR 'influenza'/exp OR 'influenza' OR 'flu'/exp OR 'flu' OR 'respiratory tract infection'/exp OR 'respiratory tract infection' OR 'influenza-like illness'/exp OR 'influenza-like illness') AND ('acute myocardial infarction'/exp OR 'acute myocardial infarction' OR 'myocardial infarction'/exp OR 'myocardial infarction' OR 'cardiovascular events' OR 'cardiovascular outcome'/exp OR 'cardiovascular outcome') | 8394 |

**Table S2.** Main characteristics of included studies evaluating the association between influenza infection and acute myocardial infraction.

| **Authors** | **Country** | **Study design** | **Type of influenza definition** | **mean age case** | **mean age Control** | **Diagnosis method of Influenza** | **Number of cases** | **Number of outcomes** | **Number of controls** | **Number of outcomes** | **Risk of bias** | **Crude OR** | **Adjusted OR (if available)** | **Adjusted OR covariate** |
| --- | --- | --- | --- | --- | --- | --- | --- | --- | --- | --- | --- | --- | --- | --- |
| Ponka et al. (1981) | Finland | Prospective hospital-based study | Laboratory-confirmed Influenza | 63 | 68 | laboratory testing (Serological test for influenza A) | 49 | 3 | 37 | 4 | High | 0.54 (0.11-2.57) |  |  |
| Guan et al. (2012) | China | Prospective hospital-based study | Laboratory-confirmed Influenza | 57.29 | 55.54 | laboratory testing (Serological test for influenza A) | 102 | 88 | 150 | 100 | Medium | 3.1 (1.5-6.4) | 5.5 (1.3–23.0) | Demographics, Risk factors of CAD, Serobiochemical markers, Antibodies to other infectious agents |
| Guan et al. (2012) | China | Prospective hospital-based study | Laboratory-confirmed Influenza | 57.29 | 55.54 | laboratory testing (Serological test for Influenza B) | 102 | 78 | 150 | 45 | Medium | 10.2 (5.7-20.0) | 20.3 (5.6–40.8) | Demographics, Risk factors of CAD, Serobiochemical markers, Antibodies to other infectious agents |
| Macintyre et al. (2013) | Australia | Prospective hospital-based study | Laboratory-confirmed Influenza | ≥40 | ≥40 | laboratory testing | 275 | 34 | 284 | 19 | Low | 1.9 (1.09-3.54) | 1.07 (0.53–2.19) | Demographics, Influenza vaccination, current smoking and high cholesterol |
| Warren-Gash et al. (2013) | England | Prospective hospital-based study | Laboratory-confirmed Influenza | 63.6 | 63.6 | Influenza A IgA antibodies | 70 | 25 | 64 | 28 | Low | 0.71 (0.33 - 1.53) | 0.82 (0.34– 2.00) | Demographics, Influenza vaccination, personal and family history of AMI |
| Abdul Aleem et al. (2023) | Bangladesh | Retrospective case-control | Laboratory-confirmed Influenza | >40 | >40 | qRT-PCR | 150 | 7 | 90 | 3 | Low |  | 2.59 (0.50–13.36) | Variable adjusted for in the final model: age, gender, tobacco use status, physical activity level, history of hypertension, diabetes, high blood cholesterol, and body mass index (BMI). |
| Matilla et al. (1989) | Finland | Prospective hospital-based study | Influenza-like illness | 44.5 | 40.5 | International Classification of Disease Codes | 40 | 11 | 41 | 4 | High |  |  |  |
| Ponka et al. (1981) | Finland | Prospective hospital-based study | Influenza-like illness | 63 | 68 | International Classification of Disease Codes | 49 | 6 | 37 | 4 | High | 1.15 (0.30 - 4.41) |  |  |
| Warren-Gash et al. (2013) | England | Prospective hospital-based study | Influenza-like illness | >40 | >40 | International Classification of Disease Codes | 71 | 10 | 64 | 3 | Low | 3.39 (0.89 - 12.92) | 3.17 (0.61– 16.47) | Influenza vaccination, personal and family history of myocardial infarction |
| Cheng et al. (2022) | Hong Kong | Retrospective case-control | Influenza-like illness | 56.4 | 56.3 | International Classification of Disease Codes | 8,840 | 41 | 21,817 | 40 | Low | 2.54 (1.64–3.92) | 1.81 (1.11–2.95) | adjusted for age, sex, medical history of hypertension, diabetes, dyslipidaemia, and atrial fibrillation/atrial flutter, period (before or after April 2009) and season (winter: November to March; non-winter: April to October). |
| Mohseni et al. (2024) | Iran | Retrospective case-control | Influenza-like illness | >50 | >50 | International Classification of Disease Codes | 150 | 111 | 150 | 39 | Moderate | 8.43 (3.44-20.62) | 3.04 (1.02–9.09) | adjusted for age, sex, medical history of infection |
| Yong and Xu (2022) | China | Retrospective case-control | Influenza-like illness | 40-75 | 40-75 | International Classification of Disease Codes | 47 | 22 | 49 | 7 | Moderate | 3.538 |  |  |

**Table S3.** **Main characteristics of included studies evaluating the incidence rate of acute myocardial infraction after influenza infection.**

| **Authors** | **Study period** | **Country** | **mean age** | **Gender ratio (male)%** | **Number of participants** | **Number of episodes** | **IRR (days 1-7)** | **IRR (days 1-3)** | **IRR (days 4-7)** | **IRR (days 8-14)** | **IRR (days 15-28)** | **IRR (days 29-91)** | **IRR (days 1-28)** | **Adjustment** | **Risk of bias** |
| --- | --- | --- | --- | --- | --- | --- | --- | --- | --- | --- | --- | --- | --- | --- | --- |
| Warren-Gash et al. 2012 | 2003-2009 | United Kingdom | 73.1 | 60.1 | 11208 | 410 |  | 7.31 (2.72–19.64) | 1.37 (.19–9.74) | 1.56 (.39–6.28) | 0.79 (.20–3.16) | 1.17 (.67–2.05) |  | Age- and Season | Low |
| Warren-Gash et al. 2018 | 2004-2014 | Scotland | 68 | 61.2 | 1227 | 179 |  | 9.80 (2.37–40.5) | 3.98 (0.55–28.9) | 2.72 (0.38–19.5) | 2.77 (0.68–11.2) |  |  | Age- and Season | Low |
| Kwong et al. 2018 | 2009-2014 | Canada | 77 | 52 | 332 | 364 | 6.05 (3.86–9.50) | 6.30 (3.25–12.22) | 5.78 (3.17–10.53) | 0.60 (0.15–2.41) | 0.75 (0.31–1.81) |  |  |  | Low |
| de Boer et al. 2024 | 2008-2019 | Netherland | 74 | 64 | 401 | 406 | 6.16 (4.11–9.24) | 6.68 (3.76–11.86) | 5.48 (3.15–9.53) | 1.96 (0.97–3.95) | 1.90 (1.13–3.18) |  | 3.18 (2.35–4.3) |  | Low |
| Korves et al. 2024 | 2010-2018 | United States | 70 | 94 | 2148 | 136 | 7.0 (5.9–8.3) |  |  | 2 (1.4–2.7) | 1.8 (1.3–2.4) |  | 3.6 (3.1–4.1) |  | Low |

**Table S4.** **Main characteristics of included studies evaluating the outcomes of acute myocardial infraction in patients with influenza infection.**

| **Authors** | **AMI patients without influenza** | **In-hospital mortality** | **Cardiogenic shock** | **Acute respiratory failure** | **Acute respiratory failure requiring mechanical ventilation** | **Acute kidney injury** | **Acute kidney injury requiring dialysis** | **Length of stay, median (DAYS)** | **Hospital costs, median** | **AMI patients with influenza** | **In-hospital mortality** | **Cardiogenic shock** | **Acute respiratory failure** | **Acute respiratory failure requiring mechanical ventilation** | **Acute kidney injury** | **Acute kidney injury requiring dialysis** | **Length of stay, median** | **Hospital costs, median** |
| --- | --- | --- | --- | --- | --- | --- | --- | --- | --- | --- | --- | --- | --- | --- | --- | --- | --- | --- |
| Cardoso et al. 2020 | 4,272,811 | 389,296 | 237,582 | 854,788 | 542,928 | 982,065 | 46,927 | 4 (2-7) | 15339 (8842-25,024) | 12,830 | 1,791 | 697 | 6,623 | 4,420 | 5,105 | 494 | 7 (4-11) | 15856 (9,144−29,891) |
| Tripathi et al. | 2,425,355 | 135820 | 135819 | 540,854 | 147947 | 366229 |  | 5.2 ± 0.01 | 21200 ± 21,3 | 3006 | 231 | 246 | 1503 | 366 | 953 |  | 8.3 ± 0.20 | 25500 ± 770 |
| Vejpongsa et al. 2019 | 1,863,615 | 159,790 | 189,495 | 416,290 |  | 450,625 | 32,075 | 5.8 ± 7.3 |  | 9885 | 1,305 | 1,895 | 5,255 |  | 3,985 | 480 | 9.3 ± 10.3 |  |
